# Supplementary material for: The interactome of intact mitochondria by cross-linking mass spectrometry provides evidence for coexisting respiratory supercomplexes
Source: Mol Cell Proteomics. 2017 Dec 8;17(2):216–32. doi: 10.1074/mcp.RA117.000470 (PMC5795388; doi:10.1074/mcp.RA117.000470)
Supplement: Supplemental Data [file supp_RA117.000470_133922_0_supp_23978_fzffwf.zip › spectra_annotation/mito_DR_spectra_annotation/343-1-7-1-14-1.pdf]

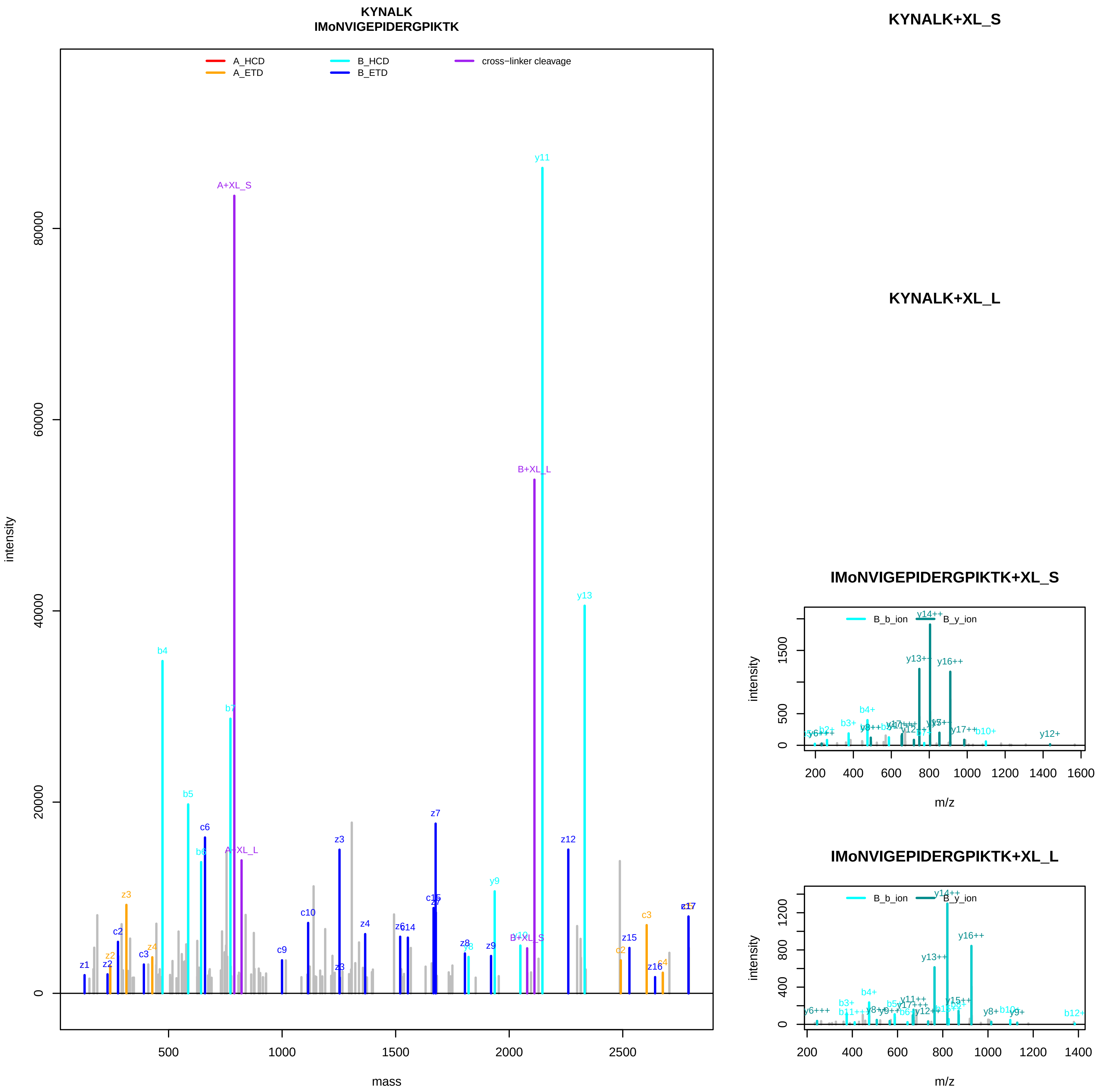

KYNALK
IMoNVIGEPIDERGPIKTK
A_HCD
A_ETD
B_HCD
B_ETD
cross−linker cleavage
intensity
mass
0
20000
40000
60000
80000
500
1000
1500
2000
2500
A+XL_S
y11
B+XL_L
y13
b4
b7
b5
c6
b6
A+XL_L
z7
z3
z12
y9
z3
c10
c9
z4
z6
c14
c15
z8
z9
y8
B+XL_S
z15
z16
c3
c2
c4
z17
z1
z2
c2
c3
z2
z3
z4
KYNALK+XL_S
KYNALK+XL_L
IMoNVIGEPIDERGPIKTK+XL_S
B_b_ion
B_y_ion
y14++
y13++
y16++
b4+
b3+
y17++
b10+
y12+
m/z
IMoNVIGEPIDERGPIKTK+XL_L
y14++
y16++
y13++
b4+
b3+
y11++
y15++
y8+
b12+
